# Patients with COVID-19 in 19 ICUs in Wuhan, China: a cross-sectional study

**DOI:** 10.1186/s13054-020-02939-x

**Published:** 2020-05-14

**Authors:** Yuan Yu, Dan Xu, Shouzhi Fu, Jun Zhang, Xiaobo Yang, Liang Xu, Jiqian Xu, Yongran Wu, Chaolin Huang, Yaqi Ouyang, Luyu Yang, Minghao Fang, Hongwen Xiao, Jing Ma, Wei Zhu, Song Hu, Quan Hu, Daoyin Ding, Ming Hu, Guochao Zhu, Weijiang Xu, Jun Guo, Jinglong Xu, Haitao Yuan, Bin Zhang, Zhui Yu, Dechang Chen, Shiying Yuan, You Shang

**Affiliations:** 1 Department of Critical Care Medicine, Union Hospital, Tongji Medical College, Huazhong University of Science and Technology, Wuhan, China; 2 Department of Intensive Care Unit, Wuhan Third Hospital, Wuhan, China; 3 Department of Critical Care Medicine, Wuhan Hospital of Traditional Chinese Medicine, Wuhan, China; 4 Department of Critical Care Medicine, Wuhan Wuchang Hospital, Wuhan, China; 5 Jinyintan Hospital, Wuhan, China; 6 Department of Emergency Internal Medicine, Tongji Hospital, Tongji Medical College, Huazhong University of Science and Technology, Wuhan, China; 7 Intensive Care Unit, Xiehe Wuhan Red Cross Hospital, Wuhan, China; 8 Department of Intensive Care Unit, Liyuan Hospital Affiliated to Tongji Medical College of Huazhong University of Science and Technology, Wuhan, China; 9 Intensive Care Unit, Tianyou Hospital Affiliated to Wuhan University of Science and Technology, Wuhan, China; 10 Intensive Care Unit, Fifth Hospital in Wuhan, Wuhan, China; 11 Intensive Care Unit, The First People’s Hospital of Jiangxia District, Wuhan, China; 12 Department of Critical Care Medicine, Wuhan Pulmonary Hospital, Wuhan, China; 13 Department of Critical Care Medicine, The Affiliated Hospital of Jianghan University, Wuhan, China; 14 Department of Critical Care Medicine, The Central Hospital of Wuhan, Tongji Medical College, Huazhong University of Science and Technology, Wuhan, China; 15 Intensive Care Unit, Union Jiangbei Hospital, Huazhong University of Science and Technology, Wuhan, China; 16 Intensive Care Unit, Dongxi Lake District People’s Hospital, Wuhan, China; 17 Department of Intensive Care Unit, The Second People’s Hospital of Three Gorges University, Yichang, China; 18 Department of Critical Care Medicine, Renmin Hospital of Wuhan University, Wuhan, China; 19 Department of Critical Care Medicine, Ruijin Hospital, Shanghai Jiao Tong University School of Medicine, Shanghai, China

**Keywords:** COVID-19, Critically ill patients, Complications, Epidemic

## Abstract

**Background:**

A COVID-19 outbreak started in Wuhan, China, last December and now has become a global pandemic. The clinical information in caring of critically ill patients with COVID-19 needs to be shared timely, especially under the situations that there is still a largely ongoing spread of COVID-19 in many countries.

**Methods:**

A multicenter prospective observational study investigated all the COVID-19 patients received in 19 ICUs of 16 hospitals in Wuhan, China, over 24 h between 8 AM February 2h and 8 AM February 27, 2020. The demographic information, clinical characteristics, vital signs, complications, laboratory values, and clinical managements of the patients were studied.

**Results:**

A total of 226 patients were included. Their median (interquartile range, IQR) age was 64 (57–70) years, and 139 (61.5%) patients were male. The duration from the date of ICU admission to the study date was 11 (5–17) days, and the duration from onset of symptoms to the study date was 31 (24–36) days. Among all the patients, 155 (68.6%) had at least one coexisting disease, and their sequential organ failure assessment score was 4 (2–8). Organ function damages were found in most of the patients: ARDS in 161 (71.2%) patients, septic shock in 34 (15.0%) patients, acute kidney injury occurred in 57 (25.2%) patients, cardiac injury in 61 (27.0%) patients, and lymphocytopenia in 160 (70.8%) patients. Of all the studied patients, 85 (37.6%) received invasive mechanical ventilation, including 14 (6.2%) treated with extracorporeal membrane oxygenation (ECMO) at the same time, 20 (8.8%) received noninvasive mechanical ventilation, and 24 (10.6%) received continuous renal replacement therapy. By April 9, 2020, 87 (38.5%) patients were deceased and 15 (6.7%) were still in the hospital.

**Conclusions:**

Critically ill patients with COVID-19 are associated with a higher risk of severe complications and need to receive an intensive level of treatments. COVID-19 poses a great strain on critical care resources in hospitals.

**Trial registration:**

Chinese Clinical Trial Registry, ChiCTR2000030164. Registered on February 24, 2020, http://www.chictr.org.cn/edit.aspx?pid=49983&htm=4

## Background

In December 2019, a series of patients in Wuhan, China, showed pneumonia-related symptoms and later being diagnosed as a novel coronavirus-caused infectious disease (COVID-19) that marks the outbreak of the epidemic [1–3]. The spread of the virus is an emerging, rapidly evolving situation and had been declared as a global pandemic by the WHO since March 11, 2020. As of March 15, 2020, there were 153,517 cases being identified worldwide [4], with 50,003 cases from Wuhan [5]. The COVID-19 pandemic poses enormous burdens and challenges to the medical care system, including intensive care units (ICUs), across different countries [6]. The higher mortality of critically ill patients was reported to be associated with the severity of the shortage of healthcare resources [7].

Previously published studies in describing the epidemiological findings, clinical presentation, and clinical outcomes of the COVID-19 patients were mainly on non-critical patients [8–10]. To our knowledge, there is only one study that was conducted with critical patients at an early time of the epidemic, which was further limited in the small sample size for the analysis [11]. In addition, all those abovementioned were retrospective studies that may be associated with possible biases or misclassifications due to the nature of retrospective looking. We conducted a multicenter 1-day cross-sectional study on critically ill patients with COVID-19 in 19 local ICUs in Wuhan. Our objective was to elaborate on the outcomes and complications of patients with COVID-19 and the intensity of treatments these patients had received.

## Methods

### Study design

This cross-sectional study was a multicenter, prospective, observational study, in which the study subjects are the patients who were received over 1 day, from 8 AM February 26, 2020, to 8 AM the next day. The involved 19 ICUs are from 16 hospitals that are designated solely for treating COVID-19 patients in Wuhan since the outbreak. There were two coordinative physicians from each of the ICU site joining the study team, who had at least a 3-year ICU working experience. All the ICUs have met the following criteria: having closed adult units, at least 10 beds, and staffed by full-time intensive care physicians and nurses covering 24 h for 7 days. Using a web-based case report form (CRF), after two rounds of pilot testing and modification, each ICU was able to perform a password-protected login to the CRF through a mobile phone connection.

All the patients in these ICUs, who were diagnosed with COVID-19 according to the Fifth Edition of Diagnosis and Treatment Protocols for Patients with Novel Coronavirus Pneumonia released by the National Health Commission of China, were registered into the study. No formal exclusion criteria were planned, and all patients’ identifiable information had been de-identified before being stored and analyzed. The study was registered in the Chinese Clinical Trial Registry (ChiCTR2000030164).

### The criteria for ICU admission

Patients were admitted to the ICUs if they met one of the following criteria: a respiratory rate of more than 40 breaths per minute, a pressure of arterial oxygen less than 60 mmHg or pulse oxygen saturation less than 90% while the patient was breathing oxygen at a flow rate of 7 L per minute or more for at least 30 min, a pressure of arterial carbon dioxide higher than 50 mmHg, hemodynamic instability and use of vasopressors, a Glasgow Coma Scale score of 12 points or lower, and need of continuous renal replacement therapy (CRRT).

### Data collection and definitions

The coordinative physicians at each site were responsible for collecting the following data from the study patients: (1) demographic information, including gender, age, pregnancy yes/no if female, occupation, date of onset of symptoms, and date of admission to ICU; (2) comorbidities; (3) vital signs and complications; (4) results of laboratory test on the study date; (5) major treatments; and (6) outcomes. The living status of all patients was followed up by April 9, 2020. If there were questions or uncertainties in the collection, the physicians went to talk to the patients’ primary care doctors for the answer or the best judgment.

In the study, acute respiratory distress syndrome (ARDS) was defined according to the Berlin definition [12], septic shock was defined according to the Sepsis-3 criteria [13], and acute kidney injury (AKI) was defined according to the KDIGO criteria [14]. Cardiac injury was defined as the hs-TnI > 28 ng/L or TnI > 0.3 ng/mL.

### Family information and visitation policies

The National Health Commission of China released a statement to classify COVID-19 as a category B infectious disease under the law on prevention and control of infectious diseases but take preventive and control measures of category A infectious diseases. COVID-19 was put under quarantinable infectious disease management according to the Frontier Health and Quarantine Law. Wuhan city was locked down on January 23, 2020; citizens included the family of COVID-19 patients who were asked to stay at home and not go out if not necessary. All the hospitalization patients are not allowed to be visited.

### Statistical analysis

We expressed descriptive data as median (with interquartile range) for continuous variables and count (%) for categorical variables. All analyses were carried out using the Stata/IC 15·1 software (StataCorp, College Station, TX, USA).

## Results

### Demographic and clinical characteristics

Data were collected from 226 patients. The number of xpatients included per ICU was 9 (7–19). Of all the patients, 217 (96.0%) were admitted to ICUs before 8 AM on February 26, 2020, and the remaining 9 (4.0%) patients were admitted to ICUs during the study period. Their age was 64 (57–70) years; 139 (61.5%) patients were male, and 22 (9.7%) patients were medical workers (Table 1). No female patients were during pregnancy. The duration from the time of ICU admission to the study date was 11 (5–17) days, and the duration from the onset of symptoms to the study date was 31 (24–36) days. Among all the patients, 155 (68.6%) had at least one coexisting disease. The common comorbidities were hypertension 96 (42.5%), diabetes 47 (20.8%), coronary heart disease 22 (9.7%), cerebrovascular disease 15 (6.6%), and chronic pulmonary disease 15 (6.6%). Twelve (5.3%) patients refused endotracheal intubation, and 11 (4.9%) patients declared do-not-resuscitate.

**Table 1:** Demographics, clinical characteristics, and clinical outcomes of 226 patients with Coivd-19 in ICUs

Characteristics	All patients (*n* = 226)
Age, years	64 (57–70)
Gender	
Male	139 (61.5%)
Female	87 (38.5%)
Occupation	
Medical worker	22 (9.7%)
Unprotected exposure history	22 (100%)
Non-medical worker	204 (90.3%)
Newly admitted to ICU	9 (4.0%)
Duration from the onset of symptom to the current study, days	31 (24–36)
Duration from ICU admission to the current study, days	11 (5–17)
Comorbidities	155 (68.6%)
Hypertension	96 (42.5%)
Coronary heart disease	22 (9.7%)
Myocardial infarction	6 (2.7%)
Congestive heart failure	4 (1.8%)
Diabetes	47 (20.8%)
Diabetes with organ damage	10 (4.4%)
Diabetes without organ damage	37 (16.4%)
Cerebrovascular disease	15 (6.6%)
Chronic pulmonary disease	15 (6.6%)
Chronic hepatopathy	3 (1.3%)
Chronic nephrosis (without regular dialysis)	3 (1.3%)
Chronic nephrosis (with regular dialysis)	5 (2.2%)
Chronic peptic ulcer	4 (1.8%)
Connective tissue disease	1 (0.4%)
Hemiplegia	4 (1.8%)
Alzheimer’s disease	4 (1.8%)
Leukemia or lymphoma	1 (0.4%)
Malignancy tumor	10 (4.4%)
Receive radiotherapy, chemotherapy, and long-term or high-dose corticoid therapy	1 (0.4%)
Refusal of endotracheal intubation	12 (5.3%)
Declaration of do-not-resuscitate	11 (4.9%)
Clinical outcome	
Remained in ICU	204 (90.3%)
Discharged from ICU	13 (5.7%)
Died	9 (4.0%)

Data are expressed as median (interquartile range) or count (%)

*COVID-19* coronavirus disease 2019, *ICU* intensive care unit

### Vital signs, complications, and laboratory tests

The vital signs in Table 2 show nothing notable, but there were patients with dysrhythmia, including 18 (8.0%) with atrial fibrillation, 2 (0.9%) with supraventricular tachycardia, and 1 (0.4%) with ventricular tachycardia.

**Table 2:** Vital signs and complications of 226 patients with COVID-19 in ICUs

Characteristics	All patients (*n* = 226)
Heart rate (bpm)	90 (76–103)
Heart rate > 125	9 (4.0%)
Systolic blood pressure (mmHg)	125 (110–137)
Systolic blood pressure < 90	2 (0.9%)
Diastolic blood pressure (mmHg)	72 (64–80)
Respiratory rate (breaths per minute)	22 (20–26)
Respiratory rate > 24	86 (38.1%)
Saturation of pulse oxygen	97 (95–99)
Saturation of pulse oxygen < 90%	14 (6.2%)
Temperature (°C)	36.7 (36.4–37)
> 37.3 to ≤ 38	21 (9.3%)
> 38	24 (10.6%)
SOFA score (*n* = 192)	4 (2–8)
ARDS	161 (71.2%)
Mild ARDS	35 (15.5%)
Moderate ARDS	47 (20.8%)
Severe ARDS	79 (35.0%)
Shock	36 (15.9%)
Septic shock	33 (14.6%)
Cardiogenic shock	2 (0.9%)
Septic combined cardiogenic shock	1 (0.4%)
Cardiac injury (hs-TnI > 28 ng/L or TnI > 0.3 ng/mL)	61 (27.0%)
Arrhythmia	21 (9.3%)
Atrial fibrillation	18 (8.0%)
Supraventricular tachycardia	2 (0.9%)
Ventricular tachycardia	1 (0.4%)
Acute kidney injury by KDIGO criteria	57 (25.2%)
Stage 1	23 (10.2%)
Stage 2	12 (5.3%)
Stage 3	22 (9.7)
Hospital-acquired bacterial or fungal infection	49 (21.7%)
Duration from the onset of symptom to the current study, days	33 (27–37)
Duration from ICU admission to the current study, days	13 (8–17.5)
Infectious foci	
Pulmonary	45 (19.9%)
Pulmonary and bloodstream	2 (0.9%)
Pulmonary and deep soft tissue	1 (0.4%)
Urinary tract	1 (0.4%)
Pneumothorax	1 (0.4%)
Gastrointestinal hemorrhage	7 (3.1%)

Data are expressed as median (interquartile range) or count (%). *N* = 226 unless specified otherwise

*COVID-19* coronavirus disease 2019, *ICU* intensive care unit, *SOFA score*, sequential organ failure estimation score, *ARDS* acute respiratory distress syndrome, *KDIGO* Kidney Disease: Improving Global Outcomes

The sequential organ failure assessment (SOFA) score was 4 (2–8). Organ function damages occurred in most of the patients: ARDS occurred in 161 (71.2%) patients, including 35 (15.5%) patients with mild ARDS, 47 (20.8%) with moderate ARDS, and 79 (35.0%) with severe ARDS. Shock occurred in 36 (15.9%) patients, including septic shock in 34 (15.0%) patients and cardiogenic shock in 3 (1.3%) patients. Cardiac injury occurred in 61 (27.0%) patients. AKI occurred in 57 (25.2%) patients, including 23 (10.2%), 12 (5.3%), and 22 (9.7%) patients with AKI of stage 1, stage 2, and stage 3, respectively.

**Fig. 1:**
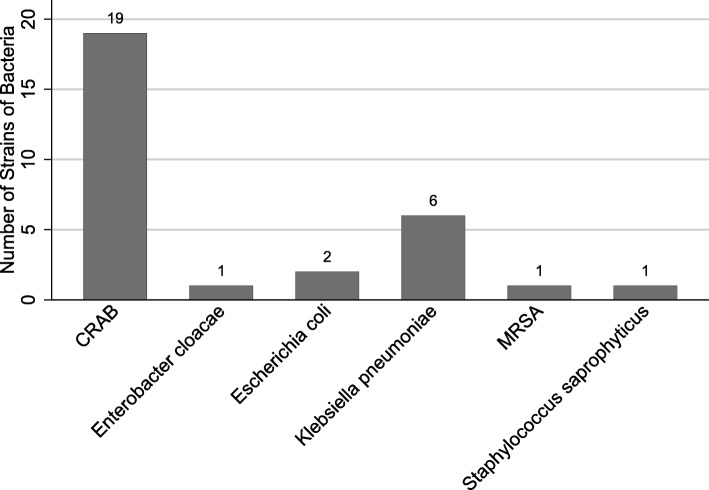
Identified bacteria in patients with hospital-acquired pneumonia. CRAB, carbapenem-resistant *Acinetobacter baumannii*; MRSA, methicillin-resistant *Staphylococcus aureus*

Hospital-acquired infections were identified in 49 (21.7%) patients. Of these patients, 1 (2.0%) patient had urinary tract infection. The remaining 48 (98.0%) patients were diagnosed with hospital-acquired pneumonia, including 2 patients and 1 patient having concomitant bloodstream infections and deep soft tissue infection, respectively. In 17 patients, the identifications of bacteria were pending. In 4 patients, carbapenem-resistant Enterobacteriaceae were entered into our web-based CRF. A total of 30 strains of bacteria were identified (Fig. 1) in the remaining 27 patients, including 3 patients with two kinds of bacteria in each of them. Among the 6 strains of *Klebsiella pneumonia*, 2 were resistant to carbapenems and 2 were positive for extended-spectrum β-lactamase.

### Laboratory tests (Table 3)

Lymphocytopenia occurred in 160 (70.8%) patients. Prolonged prothrombin time and activated partial thromboplastin time were observed from 30 (13.4%) and 51 (22.8%) patients, respectively. Elevated levels of glutamic pyruvic transaminase, glutamic oxalacetic transaminase, creatinine, and blood urea nitrogen were identified in 85 (37.6%), 46 (20.4%), 70 (31.0%), and 140 (61.9%) patients, respectively. Out of the 212 patients who had tests of D-dimer, elevated levels of D-dimer were identified in 189 (89.1%) patients. For 162 patients who underwent tests on serum myoglobin, excessive myoglobin level was identified from 57 (35.2%) patients, with the level higher than 1000 ng/mL in 10 (6.2%) patients.

**Table 3:** Laboratory findings of 226 patients with COVID-19 in ICUs

Characteristics	All patients (*n* = 226)
Blood routine	
White blood cell count (×10^9^ per L; normal range 4–10)	8.54 (5.89–12.69)
Increased	95 (42.0%)
Decreased	16 (7.1%)
Hematocrit (%)	31.1 (26.3–35.7)
Hemoglobin (g/L; normal range 130–175)	98 (85–116)
Decreased	220 (97.3%)
Neutrophils (×10^9^ per L; normal range 1.8–6.3)	7.28 (4.24–10.94)
Increased	127 (56.2%)
Decreased	4 (1.8%)
Lymphocytes (×10^9^ per L; normal range 1.1–3.2)	0.84 (0.56–1.19)
Increased	1 (0.4%)
Decreased	160 (70.8%)
Platelets (×10^9^ per L; normal range 125–350)	181.5 (115–258)
Increased	14 (6.2%)
Decreased	66 (29.2%)
Coagulation panel (*n* = 223)	
Prothrombin time (s; normal range 11–16)	13 (11.6–14.7)
> 16 to ≤ 19	19 (8.5%)
> 19	11 (4.9%)
Activated partial thromboplastin time (s; normal range 28–43.5)	32.3 (26.1–42.1)
> 43.5 to ≤ 48.5	17 (7.6%)
> 48.5	34 (15.2%)
D-dimer (*n* = 212) (mg/L; normal range < 0.5)	3 (1.2–7.1)
> 0.5 to ≤ 1	20 (9.4%)
> 1	169 (79.7%)
Hepatic function	
Glutamic pyruvic transaminase (U/L; normal range < 40)	31.2 (19–57)
Increased	85 (37.6%)
Glutamic oxalacetic transaminase (U/L; normal range < 50v)	31.6 (22–48)
Increased	46 (20.4%)
Total bilirubin concentration (μmol/L, normal range 3–22)	12.6 (8.6–19.1)
Increased	42 (18.6%)
Albumin concentration (g/L, normal range 35–50)	32.8 (29.4–36.9)
Decreased	145 (64.2%)
Renal function	
Serum creatinine concentration (μmol/L, normal range 46–92)	64.2 (49–111.6)
Increased	70 (31.0%)
Blood urea nitrogen (mmol/L, normal range 2.5–6.1)	7.34 (5.2–14.1)
Increased	140 (61.9%)
Serum electrolyte	
Potassium (mmol/L, normal range 3.5–5.1)	4.1 (3.7–4.56)
Sodium (mmol/L, normal range 135–145)	140 (137–144)
Myoglobin plasma concentration (*n* = 162) (ng/mL, normal range < 150)	
< 150	105 (64.8%)
≥ 150 to < 1000	47 (29.0%)
≥ 1000	10 (6.2%)
Procalcitonin (*n* = 220) (ng/mL, normal range < 0.05)	0.19 (0.05–1.4)
< 0.05	58 (26.4%)
0.05–0.5	80 (36.4%)
> 0.5	82 (37.3%)
Ferritin concentration (*n* = 122) (μg/L, normal range < 500)	
< 500	31 (25.4%)
≥ 500 to < 1000	33 (27.1%)
≥ 1000 to < 1500	17 (13.9%)
≥ 1500 to < 2000	10 (8.2%)
≥ 2000	31 (25.4%)

Data are expressed as median (interquartile range) or count (%). *N* = 226 unless specified otherwise

*COVID-19* coronavirus disease 2019, *ICU* intensive care unit

### Managements (Table 4)

Of all the patients, 85 (37.6%) received invasive mechanical ventilation, with 14 (6.2%) treated with extracorporeal membrane oxygenation (ECMO) at the same time and 20 (8.8%) received noninvasive mechanical ventilation. Prone position ventilation was conducted in 22 (9.7%) patients and continuous renal replacement therapy (CRRT) in 24 (10.6%) patients. Spontaneous breathing test was conducted in 17 (7.5%) patients, with 15 (6.6%) failed and 2 (0.9%) passed, and one (0.4%) patient who passed the test was extubated.

**Table 4:** Managements

Variables	All patients (*n* = 226)
Respiratory support	
None	11 (4.9%)
Oxygen delivery by nasal cannula	59 (26.1%)
Oxygen delivery by mask	14 (6.2%)
High-flow nasal cannula (HFNC)	37 (16.4%)
Noninvasive mechanical ventilation	20 (8.8%)
Invasive mechanical ventilation	85 (37.6%)
ECMO	14 (6.2%)
Prone position	22 (9.7%)
Continuous renal replacement therapy	24 (10.6%)
Vasoactive drugs	48 (21.2%)
Intravenous antihypertensive drugs	14 (6.2%)
Central venous catheterization	22 (9.7%)
Thoracic cavity closed drainage	1 (0.4%)
Accidental removal of tracheal tube	2 (0.9%)
Spontaneous breathing test	17 (7.5%)
Spontaneous breathing test (failed)	15 (6.6%)
Spontaneous breathing test (passed)	2 (0.9%)
Removal of tracheal tube	1 (0.4%)
Chest imaging examination	56 (24.8%)
Ultrasound examination	63 (27.9%)
Chest or lung ultrasound examination	52 (23.0%)
Antivirus agent	117 (51.8%)
Ribavirin	35 (15.49%)
Ganciclovir	6 (2.65%)
Interferon inhalation	9 (3.98%)
Arbidol	51 (22.57%)
Lopinavir-ritonavir	12 (5.31%)
Neuaminidase inhibitors	10 (4.42%)
Thymosin	92 (40.7%)
Antimicrobial agents	168 (74.3%)
Systemic glucocorticoids	37 (16.4%)
Immunoglobulin	29 (12.8%)
Blood transfusion	19 (8.4%)
Red blood cell	11 (4.9%)
Plasma	9 (4.0%)
Blood platelet	1 (0.4%)
Traditional Chinese herb	59 (26.1%)

Data are *n* (%) unless specified otherwise

**Fig. 2:**
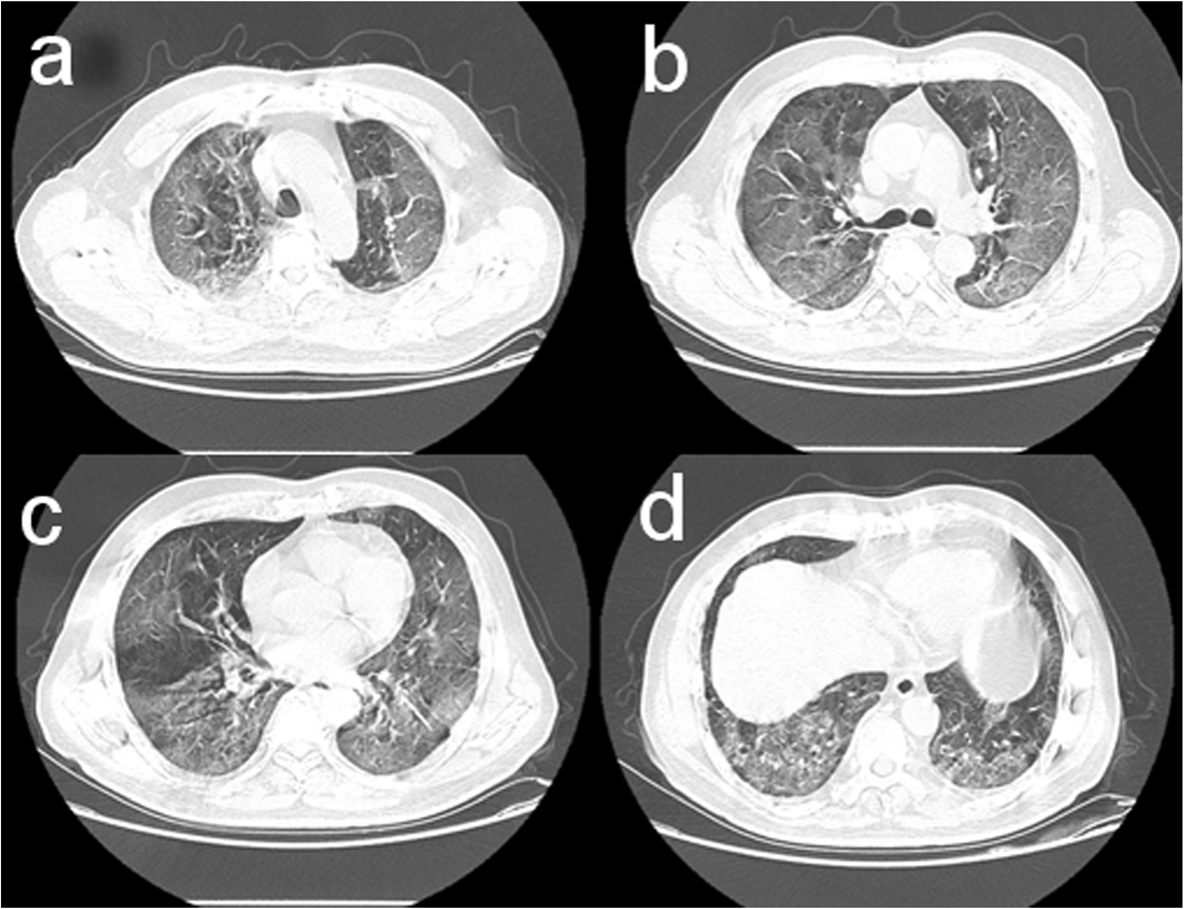
Chest computed tomographic imaging of the lungs performed on February 26, 2020, showed ground-glass opacity in the bilateral lungs on day 10 after symptom onset

Fifty-six (24.8%) patients received chest radiological examinations including chest computed tomography and X-ray; all the patients showed a bilateral lesion of the lungs (Fig. 2). Sixty-three (27.9%) received an ultrasound examination, including 52 (23.0%) chest or lung ultrasound examinations.

As for medications, 117 (51.8%) patients received antivirus agents, 168 (74.3%) received antimicrobial agents, 92 (40.7%) received a subcutaneous injection of thymosin, 37 (16.4%) patients received glucocorticoids intravenously, and 29 (12.8%) patients received immunoglobulin.

### Outcomes

At the end of the study, 204 (90.3%) patients remained in the ICUs, 13 (5.7%) were already discharged, and 9 (4.0%) died during the observation period. By April 9, 2020, among the 226 patients included, 87 (38.5%) patients were deceased and 15 (6.7%) were still in the hospital (Table 5).

**Table 5:** Outcomes of 226 patients with COVID-19 by April 9, 2020

Outcome	Age groups (years)						All patients (*n* = 226)
	30–40 (*n* = 7)	41–50 (n = 19)	51–60 (*n* = 51)	61–70 (*n* = 97)	71–80 (*n* = 35)	81–90 (*n* = 17)	
Still hospitalized	0 (0.0%)	2 (10.5%)	4 (7.8%)	8 (8.3%)	0 (0.0%)	1 (5.9%)	15 (6.7%)
Discharged	7 (100.0%)	8 (42.1%)	31 (60.8%)	45 (46.4%)	23 (65.7%)	10 (58.8%)	124 (54.9%)
Died	0 (0.0%)	9 (47.4%)	16 (31.4%)	44 (45.4%)	12 (34.3%)	6 (35.3%)	87 (38.5%)
Died with DNI	0 (0.0%)	0 (0.0%)	1 (2.0%)	3 (3.1%)	0 (0.0%)	3 (17.6%)	7 (3.1%)
Patients received IMV	4 (57.1%)	15 (78.9%)	25 (49.0%)	55 (56.7%)	18 (51.4%)	4 (23.5%)	121 (53.5%)
Died ever receiving IMV	0 (0.0%)	9 (47.4%)	15 (29.4%)	40 (41.2%)	12 (34.2%)	3 (17.6%)	79 (35.0%)
Received NIV before IMV	3 (42.9%)	6 (31.6%)	11 (21.6%)	29 (29.9%)	12 (34.3%)	1 (5.9%)	62 (27.4%)

Data are expressed as count (%)

*COVID-19* coronavirus disease 2019, *DNI* do not intubate, *IMV* invasive mechanical ventilation, *NIV* noninvasive ventilation

## Discussion

In this cross-sectional study on critically patients with COIVD-19, we found that ARDS occurred in 161 (71.2%), septic shock occurred in 34 (15.0%), AKI in 57 (25.2%), and cardiac injury in 61 (27%) of the 226 patients. Of all of them, 85 (37.6%) were being treated with invasive mechanical ventilation, including 14 (6.2%) on ECMO at the same time; 20 (8.8%) treated with noninvasive mechanical ventilation; and 24 (10.6%) treated with continuous renal replacement therapy. At the follow-up, 121 (53.5%) were performed invasive mechanical ventilation and 87 (38.5%) died.

To the extent of our knowledge, this study is by far the only prospective epidemiological study on critically ill patients with COVID-19. Based on the report published by the Chinese CDC, among all COVID-19 in China, 5% were categorized critically ill, i.e., with respiratory failure, septic shock, and/or multiple organ dysfunction or failure [15]. On February 26, 2020, the number of accumulated patients with COVID-19 in China was 39,755 [16], which implies a sample of more than 10% of critically ill patients in China by the time have been presented in our study. We hope the information given here will shed light on the timely update of the critically ill patient care in an ICU in this global pandemic. We want to emphasize the major finding from this study that the intensive level of treatments needs to be given to a large portion of patients. In light of the exponential growth trend of the increased number of new COVID-19 cases, the critical care resources should be on the top list of the ICU warehouse against the pandemic disease.

In our study, we found that the median age of all the patients included was 64 years, and 61.5% of the patients were male. Previous studies showed that the median age of critically ill patients with COVID-19 was 60–66 years, and 67–70% of these patients were males [8, 9, 17]; ARDS tends to occur in male patients with advanced age [18, 19]. Another earlier publication reported that of the COVID-19 patients admitted to the ICU, 61.1% were identified as ARDS, 41.7% received noninvasive ventilation, 47.2% received invasive ventilation, and 11.1% required ECMO [20]. These findings are in agreement or similar to the output of our study. However, from our data, 22 of 79 (36.7%) patients with severe ARDS received prone position ventilation, which was much higher than the 8.7% reported in a cross-sectional survey of ARDS in mainland China in 2018 which did not involve COVID-19 patients [21]. This is a small sign that manifests the differentiation in treating patients in an ICU for a general situation versus the ongoing pandemic.

We found that septic shock occurred in 15.0% of critically ill patients with COVID-19. Besides the hospital-acquired infection identified from 49 (21.7%) patients, we postulate that SARS-CoV-2 could play an important role in the development of septic shock, based on the evidence that even 31 (24–36) days after the onset of symptoms, 70.8% still had lymphocytopenia. Whether there is viremia of SARS-CoV-2 causing septic shock is difficult to determine. But the bottom line is that lymphocytopenia was associated with an increased risk of acquired infection in ICU [22]. And lymphocytopenia was proved to be associated with the probability of 28-day septic shock and 28-day mortality [23].

The rate of AKI in critically ill patients with COVID-19 was high. The pathogenesis of AKI has not been fully understood yet, but it may be associated with ACE2, the cell entry receptor of the SARS-CoV-2. It has been identified to be exclusively expressed not only in the respiratory organs, but also in other organs, for example, the kidney, which may facilitate the direct invasion and damage [24]. CRRT was used in 10.6% of our patients during the study, compared with 5% of critically ill patients during the SARS epidemic in Canada [25]. We are facing a worse situation in using CRRT now compared with the SARS outbreak.

One unexpected finding was that 57 (35.2%) critically ill patients with COVID-19 were with increased levels of myoglobin. One third of the overall COVID-19 patients were experiencing myalgia [20]. A possible explanation is that the SARS-CoV-2 might damage the muscle system. Whether the damage leads to muscle weakness and thereby causes failure of spontaneous breathing trial needs further evaluation.

Physicians treating patients with COVID-19 were under personal protective equipment, which made them impossible to perform an auscultation. Lung ultrasound was an effective technique to replace auscultation and assess the etiologies of lung abnormalities and their severity level [26]. A total of 52 (23.0%) patients received chest or lung ultrasound examinations during the period. At the same time, 56 (24.8%) patients received chest imaging examinations, which might consume more medical resources than ultrasound, especially in the isolation wards. Training more physicians capable of doing chest or lung ultrasound might reduce the dependence on chest imaging examinations.

Previous study reported different mortality rates in critically ill patients, from 16.7% [20] to 26% [27], 61.5% [11], and 67% [28]. In our study, only 6.7% of the patients were still hospitalized in the general wards or ICUs, compared with 58.3% hospitalized [20] and 58% in ICUs [27], and 23.1% hospitalized [11] and 24% in ICUs [28] in other studies, respectively. The mortality rates get higher if the follow-up time prolongs. However, the criteria for ICU admission were different among the studies, which was another reason for the different mortality rates.

Our study has some limitations. First, our study was conducted only in Wuhan, China. But on February 26, 2020, 32,392 in 39,755 (81.5%) patients being treated were in Wuhan [16]. We believe the rate of critically ill patients being treated in Wuhan was higher than 81.5%, which meant that the findings from this study could probably be generalized. Second, the selection of ICUs was not random. However, all the ICUs were selected from the hospital designated for patients with COVID-19 only, and all the ICUs were closed adult units, staffed by qualified full-time intensive care physicians and nurses for 24 h. Third, some important data, for example, arterial blood gas analysis, were not available in some patients. That was because this study was an observational study, and we intended to intervene in the routine practices of different ICUs as less as possible. Forth, the appointed physicians most likely were not the treating physicians for all the patients in their ICUs. All the directors appointed two experienced physicians for the study, who tried to eliminate the bias by clearing uncertainties with the treating physicians.

## Conclusion

Critically ill patients with COVID-19 are associated with considerable rates of severe complications and need treatments of high intensity. COVID-19 poses great strains on critical care resources in hospitals.

## Data Availability

After publication, the date will be made available to others on reasonable requests to the corresponding author. A proposal with a detailed description of the study objectives and statistical analysis plan will be needed for the evaluation of the reasonability of requests. Additional materials might also be required during the process of evaluation. Deidentified participant data will be provided after the approval of the corresponding author and Wuhan Jinyintan Hospital.

## Abbreviations

COVID-19: Coronavirus disease 2019
ICU: Intensive care unit
ARDS: Acute respiratory distress syndrome
ECMO: Extracorporeal membrane oxygenation
AKI: Acute kidney injury
KDIGO: Kidney Disease: Improving Global Outcomes
SOFA: Sequential organ failure assessment
CRRT: Continuous renal replacement therapy
CDC: Centers for disease control
